# Late Complications in Long-Term Childhood Cancer Survivors: What the Oral Health Professional Needs to Know

**DOI:** 10.3390/dj12010017

**Published:** 2024-01-19

**Authors:** Sali Al-Ansari, Juliette Stolze, Dorine Bresters, Alan Henry Brook, Alexa M. G. A. Laheij, Henk S. Brand, Göran Dahllöf, Frederik R. Rozema, Judith E. Raber-Durlacher

**Affiliations:** 1 Department of Oral Medicine, Academic Centre for Dentistry Amsterdam (ACTA), University of Amsterdam and Vrije Universiteit Amsterdam, 1081 LA Amsterdam, The Netherlands; sali.al-ansari@fachklinik-hornheide.de (S.A.-A.); a.laheij@acta.nl (A.M.G.A.L.);; 2 Department of Oral and Maxillofacial Surgery, Amsterdam UMC, University of Amsterdam, 1081 HZ Amsterdam, The Netherlands; 3 Department Maxillofacial Surgery, Fachklinik Horneide, 48157 Münster, Germany; 4 Department of Oral Biochemistry, Academic Centre for Dentistry Amsterdam (ACTA), University of Amsterdam and Vrije Universiteit Amsterdam, 1081 LA Amsterdam, The Netherlands; jstolzetandarts@gmail.com (J.S.); h.brand@acta.nl (H.S.B.); 5 Princess Máxima Center for Pediatric Oncology, 3584 CS Utrecht, The Netherlands; d.bresters@prinsesmaximacentrum.nl; 6 Department of Oral and Maxillofacial Surgery, Prosthodontics and Special Dental Care, University Medical Center Utrecht, 3584 CX Utrecht, The Netherlands; 7 Adelaide Dental School, University of Adelaide, Adelaide 5005, Australia; alan.brook@adelaide.edu.au; 8 Institute of Dentistry, Queen Mary University of London, London E12AD, UK; 9 Division of Orthodontics and Pediatric Dentistry, Karolinska Institutet, 14152 Huddinge, Sweden; goran.dahllof@ki.se; 10 Center for Oral Health Services and Research, Mid-Norway (TkMidt), 100098 Trondheim, Norway

**Keywords:** pediatric cancer, childhood cancer survivors, late effects, chronic oral complications, oral care, dental treatment, xerostomia, hyposalivation

## Abstract

With diagnostic and therapeutic advances, over 80% of children diagnosed with cancer become long-term survivors. As the number of childhood cancer survivors (CCS) continues to increase, dental practitioners become more likely to have CCS among their patients. CCS may develop late complications from damage caused by their cancer treatment to endocrine, cardiovascular, musculoskeletal, and other organ systems. These complications may surface decades after the completion of treatment. Adverse outcomes of childhood cancer treatment frequently involve oral and craniofacial structures including the dentition. Tooth development, salivary gland function, craniofacial growth, and temporomandibular joint function may be disturbed, increasing oral health risks in these individuals. Moreover, CCS are at risk of developing subsequent malignancies, which may manifest in or near the oral cavity. It is important that dental practitioners are aware of the childhood cancer history of their patients and have knowledge of potential late complications. Therefore, this narrative review aims to inform dental practitioners of late oral complications of cancer treatment modalities commonly used in pediatric oncology. Furthermore, selected common non-oral late sequelae of cancer therapy that could have an impact on oral health and on delivering dental care will be discussed.

## 1. Introduction

With advances in therapeutic strategies for common pediatric cancers such as leukemia, lymphoma, and brain and nervous system tumors, the number of childhood cancer survivors (CCS) continues to increase, particularly in high-income countries [1,2]. More than 80% of children with cancer currently survive at least 5 years post diagnosis and the majority are cured [3,4].

Nevertheless, treatment of childhood cancers with cytotoxic chemotherapy, radiotherapy, hematopoietic cell transplantation (HCT), immunotherapy, and targeted therapies or regimens using combinations of these treatment modalities is not only associated with acute complications but often leads to permanent damage to organ systems as well. This damage may give rise to late complications that only become clinically apparent after many years [5,6]. Long-term CCS are more likely to have impaired physical and psychological health status and to die prematurely compared to the general population [6,7]. Almost 75% of CCS experience one or more late adverse health effects and almost half of these late effects are severe or even life-threatening [8,9]. Late complications in CCS largely depend on the type of therapy the patient was treated with and baseline patient characteristics including age, tumor-related factors, genetic susceptibility, and lifestyle factors later in life [10,11].

Adverse outcomes of childhood cancer treatment also frequently involve oral and craniofacial structures including the dentition. Tooth development, salivary gland function, craniofacial growth, and temporomandibular joint function might be disturbed and lead to decreased oral health and oral health-related quality of life [12,13]. Furthermore, CCS are at risk for developing subsequent malignancies, which may manifest in or near the oral cavity [14,15].

While the value of involving dental professionals in the management of acute oral complications in pediatric cancer patients is well recognized [16,17], the need for follow-up oral and dental care in CCS is mainly published in the oncology literature and is probably less acknowledged by dental providers. Long-term CCS are usually under the care of general dental practitioners, who may not be aware of their patients’ cancer history and may have no knowledge of late oral adverse effects that may result from cancer treatment during childhood [18]. In addition, late effects of childhood cancer and its treatment elsewhere in the body may affect oral health and dental treatment planning.

As more dental care providers are likely to encounter CCS more frequently in their practice, this article aims to inform dental practitioners of late oral complications of cancer treatment modalities commonly used in pediatric oncology. In addition, it discusses selected non-oral late effects of cancer therapy in long-term survivors that may affect oral health and the provision of dental care.

## 2. Methods

Medline through Pubmed and the Web of Science were searched until September 2023. In addition, we screened Google Scholar and performed a manual search to screen the references of articles of interest to identify additional studies that were not retrieved in the original search. We also searched for clinical practice guidelines of professional organizations. The search was limited to literature written in the English language. The search included the following search terms: childhood cancers, pediatric cancers, (long-term) childhood cancer survivors, chemotherapy, radiation/radiotherapy, stem cell transplantation, late effects/complications, late oral effects/complications, late oral/orofacial complications, tooth development, cranial/facial growth, hyposalivation, xerostomia, caries, gingivitis, periodontitis, second primary cancer, chemotherapy, radiation, stem cell transplantation, chronic (oral) graft-versus-host disease, osteoradionecrosis, medication-related necrosis of the jaw.

## 3. Late oral and Craniofacial Complications

### 3.1. Tooth Development Disturbances

**Figure 1:**
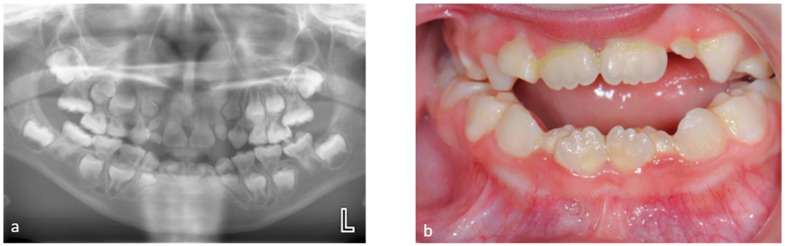
(**a**) Orthopantomogram showing the mixed dentition with extensive short and tapered roots of canines, primary and permanent first molars, and severe crowding in an 8-year-old patient. Stem cell transplant (for juvenile myelomonocytic leukemia) took place at 7 months of age. (**b**) Clinical picture of this patient. Mixed dentition showing areas of white/cream opacities on permanent central incisors and severe crowding.

**Figure 2:**
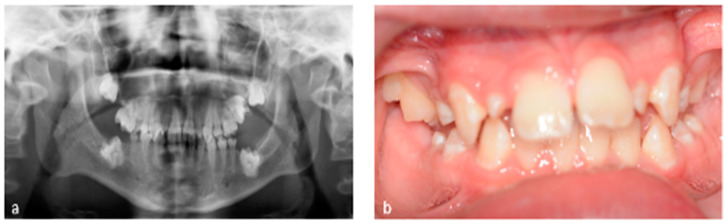
(**a**) Orthopantomogram showing the permanent dentition with multiple aplasia, microdontia, short and tapered roots, as well as impacted teeth and changes in condylar form in a 13-year-old patient. Stem cell transplant (for Hurlers syndrome) was performed at 2 years of age. (**b**) Clinical picture of this patient.

Tooth development is a complex process controlled by a sequence of cellular and molecular networks that act at particular times and places [19]. Cancer treatment during stages of tooth development can disturb these networks and lead to dental anomalies [20,21]. Examples of common tooth development anomalies and problems with occlusion are depicted in Figure 1a,b and Figure 2a,b. Antineoplastic therapies that affect the cells involved in odontogenesis may cause changes in tooth enamel and root development, premature apex closure, dental development delay, or retained teeth [22].

The incidence and severity of these anomalies is increased after oncologic treatment at ages younger than 5 years and in CCS treated with higher doses of alkylating agents [12,18,23]. Radiation therapy involving the oral cavity also increases the risk of disturbed dental development since ameloblasts can be damaged by doses as low as 10 Gy. However, in this case, the dental development disturbances will be limited to the irradiated area [12].

Dental anomalies include hypodontia, microdontia, enamel defects, root development disturbances, delayed eruption, and persistent deciduous teeth, which may lead to anatomical, functional, and esthetic disturbances and decreased quality of life [22,24,25,26,27] The prevalence of hypodontia in CCS varies between 6% and 44% and is most commonly observed in second premolars and second molars [25,28,29,30]. Studies showed a 22–78% prevalence of microdontia in CCS treated with HCT [29,31,32,33,34]. Hypodontia and microdontia can cause spacing and movement of teeth resulting in poor dental alignment. In addition to a less favorable appearance, patients may suffer from malocclusion, reduced chewing ability, temporomandibular joint complaints, periodontal attachment loss, insufficient alveolar bone growth, inarticulate pronunciation, and other problems [35].

Other common dental defects associated with chemotherapy are enamel hypoplasia, opacities, and discoloration, with a prevalence between 36% and 69.8% [22,28,36]. Fever and antibiotic use during chemotherapy treatment could play a contributing role [37]. Enamel defects may increase caries risk and may lead to esthetic problems.

Root development disturbances are also commonly identified in CCS and include shape and length malformations, as well as apical blunting [36]. Nasman et al. [38], reported that 94% of children, with a mean age of 10.8 years, treated with total body irradiation and cyclophosphamide followed by HCT had teeth with short, V-shaped roots. Short roots may lead to early tooth loss and high forces on these teeth should be avoided when planning orthodontic treatment. Moreover, apical malformations may complicate endodontic treatment.

In CCS, root development should be evaluated with panoramic radiographs prior to dental and orthodontic procedures to prevent complications [12].

### 3.2. Disturbed Craniofacial Growth

The physiology of craniofacial growth and development is complex and involves interaction between different craniofacial anatomic areas, including the dentition and the mandible, during childhood and adolescence [39]. Cranial base growth is largely completed by age 5, whereas facial growth continues during the second decade of life [40].

Radiotherapy to the growing craniofacial skeleton can cause significant clinical problems despite advances in targeting the radiation field [41,42]. Not only bone but also soft tissue, muscle, and blood vessels are sensitive to irradiation and may contribute to changes in the shape of the child’s face [43,44]. In addition to the direct effects of radiotherapy, an altered hypothalamic–pituitary function resulting in diminished growth hormone production may contribute to craniofacial growth disturbances.

Although in general the influence of chemotherapy on craniofacial growth is less extensive than that of radiotherapy [45], certain chemotherapeutic agents (e.g., methotrexate and ifosfamide) can adversely affect growth [39,46].

A statistically significant correlation between age at HCT, degree of disturbances in dental development, and vertical facial growth was found [47]. In this study, conditioning regimens including busulfan or total body irradiation had similar deleterious effects on tooth area reduction and craniofacial parameters.

Craniofacial abnormalities occurred in 90% of childhood leukemia survivors who received a combination of chemotherapy and cranial radiotherapy (24 Gy) before 5 years of age, most often manifesting as reduced mandibular growth [48]. Similar observations were reported by Dahllöf [45]. Fortunately, nowadays radiotherapy is less often indicated in leukemia [49].

Moreover, deformities can be caused directly by surgical procedures in the head and neck area and indirectly by growth and developmental disturbances resulting from this surgery.

Craniofacial deformities resulting from disturbed growth are often associated with functional and esthetic problems negatively affecting an individual’s self-image. Moreover, craniofacial deformities are often associated with other late oral sequelae such as dental anomalies, hyposalivation, and trismus [42]. Management of craniofacial deformities may be challenging and may require teamwork from multiple professionals. In children at risk of disturbed craniofacial growth, early consultation with a specialized orthodontist is important. In addition, innovative surgical reconstruction techniques tailored to the individual patient may be required [12,50,51].

### 3.3. Craniomandibular Dysfunction

Head and neck radiotherapy in particular is associated with late musculoskeletal effects and may induce long-term alterations in connective and muscle tissues, resulting in inflammation and eventually fibrosis [52,53]. These changes in tissue homeostasis and concomitant growth retardation may lead to malocclusion and reduced mobility of the temporomandibular joint, which can lead to pain and headaches.

Dahllöf et al. [54] reported that 84% of CCS who received conditioning for HCT with total body irradiation experienced craniomandibular dysfunction including trismus. In CCS treated for nasopharyngeal carcinoma with radiation doses greater than 50 Gy, the prevalence of trismus ranged from 7 to 27% [55]. Sclerosis associated with chronic oral Graft-versus-Host-Disease (cGVHD, discussed below) may also lead to trismus due to both cutaneous and mucosal fibrosis [56].

Trismus is associated with compromised oral hygiene, poor nutrition, difficulties with speech, and may complicate dental treatment. In patients at risk for trismus, early interventions including jaw-stretching exercises are crucial. A specialized oral facial physiotherapist may be included in the treatment team.

### 3.4. Salivary Gland Dysfunction

Studies on salivary gland dysfunction in long-term survivors are still scarce, and therefore the prevalence of xerostomia (the sensation of oral dryness) and/or hyposalivation (decreased saliva secretion) in CCS is likely to be underreported.

In a large multicenter study, the prevalence of xerostomia in CCS was 2.8% compared to 0.3% in their siblings, with an increased risk in survivors older than 30 years of age [57]. In a cross-sectional study in almost 300 long-term CCS, hyposalivation was identified in nearly one-third of CCS, whereas only 10% reported xerostomia [58]. Associated factors for hyposalivation were female gender and radiotherapy >12 Gy to the salivary glands. The long-term effects of relatively new treatment modalities such as intensity-modulated radiotherapy (IMRT) or proton beam radiation that allow dosimetric sparing of the salivary glands remain to be determined.

The contribution of chemotherapy alone to hyposalivation and xerostomia in CCS is not yet fully elucidated and the reported results are somewhat controversial. Avşar et al. [36] found no significant changes in salivary flow in CCS treated with chemotherapy, whereas Németh et al. [59] reported that chemotherapy decreased the output of the major salivary glands, but increased the saliva production of the minor salivary glands. Al-Ansari et al. [60] observed that chemotherapeutic agents induce changes in mice salivary glands that could affect ion transport, potentially leading to changes in the composition of saliva.

Furthermore, CCS may use multiple medications, which may negatively impact salivation [61].

The consequences of quantitative and qualitative salivary changes include oral mucosal sensitivity, increased risk of mucosal infections and dental caries, taste changes, sleep disturbance, and difficulties with chewing, swallowing, and speaking, which may all negatively impact CCS’s quality of life [62]. Therefore, regular dental check-ups and screening CCS for xerostomia and hyposalivation are important to ameliorate symptoms and prevent adverse effects on oral and overall health. Detailed information on diagnosis and management of hyposalivation and xerostomia is provided elsewhere [63,64].

### 3.5. Dental Caries and Periodontal Diseases

Studies reported a higher prevalence of caries in CCS compared to age and sex-matched controls [30,36,59,65,66,67]. Qualitative and quantitative salivary changes, a shift of the oral microbiome towards a more cariogenic microflora, and enamel defects as a result of cancer treatment and other treatment exposures are considered factors contributing to a higher risk of caries. In addition, decreased oral hygiene, dietary changes, and socioeconomic and demographic factors may play a role.

Wogelius et al. [66] reported that children diagnosed with cancer before 5 years of age did not have increased caries prevalence in their permanent dentition when evaluated at ages 12 and 15. In contrast, children diagnosed with cancer between 5 and 6 years of age had an increased prevalence of severe caries at age 12 compared to controls, but this difference disappeared by age 15. Nevertheless, those who had been treated with head and neck radiotherapy continued to have a high caries risk. Stolze et al. [18] reported that, according to their dentist, 20.4% of CCS were considered to have increased caries susceptibility. In a large retrospective study, long-term CCS reported many carious lesions (49%), but this did not differ from controls (47%) [68].

In this study of Patni et al. [68], however, 10% of the CSS reported having severe gingivitis and/or periodontitis, significantly higher than in controls (5.3%). A previous systematic review identified more gingival inflammation in CCS post-chemotherapy compared to controls [69]. Studies in adult cancer patients indicate that radiotherapy to the head and neck area deteriorates periodontal health in a dose-dependent manner as a result of decreased cellularity, vascularity, and reduced healing/remodeling potential of the periodontium [70,71,72]. Furthermore, CCS may be at increased risk of developing diabetes mellitus, which may affect periodontal health. This will be discussed in more detail below.

Although more research is needed on the prevalence of caries and periodontal diseases in CCS, it is advisable for dental practitioners to pay more attention to prevention and early diagnosis of these oral diseases in CCS [73].

### 3.6. Oral Graft-versus-Host-Disease

Chronic Graft-versus-host-disease (cGVHD) is a serious and potentially life-threatening complication of allogeneic HCT that may affect multiple organs, including the oral cavity. cGVHD occurs when immune cells transplanted from a non-identical donor (graft) into the recipient (host) recognize the host cells as “foreign”, thereby attacking these cells. cGVHD most often manifests in the first year after allogeneic HCT but may even develop several years later.

Oral cGVHD usually presents as lichenoid and ulcerative mucosal lesions [74]. Damage to salivary glands and sclerosis is less common in CCS with cGVHD compared to patients with cGVHD transplanted in adulthood [75,76]. Symptoms include mucosal sensitivity and pain and less frequently dry mouth, taste changes, and limited mouth opening. Nevertheless, it is recommended to assess salivary gland function, oral hygiene, and any dietary changes in CCS with oral cGVHD as they affect the risk of caries.

CCS with (a history of) cGVHD are especially at risk of developing oral cancer (see below) even many years after HCT [77]. Therefore, regular dental check-ups, including screening for oral cancer, are particularly important in these survivors [78]. Oral cGVHD manifestations may require referral to an oral medicine specialist and invasive dental interventions should be carried out in close consultation with the medical team [79].

### 3.7. Osteoradionecrosis and Medication-Related Osteonecrosis of the Jaw

Osteoradionecrosis (ORN) of the jaw is a complication infrequently observed in CCS treated with high-dose radiotherapy (>40 Gy) to the head and neck area. It may develop at sites with periodontitis and after invasive dental procedures [12,80]. In CCS with a history of head and neck radiotherapy, the dentist should obtain information on which structures were exposed to high-dose radiation and the implications for procedures such as extractions.

Medication-related osteonecrosis of the jaw (MRONJ) seems rare in children [81,82]. However, medications associated with MRONJ risk such as bisphosphonates, denosumab, and bevacizumab have been introduced in pediatric oncology [83]. Moreover, antiresorptive agents may be indicated for osteoporosis and osteopenia associated with pediatric cancer treatment [84]. Although the risk of developing MRONJ may be very low in pediatric populations, continued surveillance is advised [85]. Ideally, a dental assessment should be performed before starting the administration of antiresorptives or antiangiogenics to eliminate potential risk factors for MRONJ and thereafter, meticulous oral hygiene and regular dental checkups to maintain oral health are warranted.

### 3.8. Subsequent Primary Malignancies

CCS have a risk of developing subsequent primary malignancies, which may be located in the head and neck area [86]. These malignancies may develop years after pediatric cancer treatment. A large European study investigating oral cancer risk in almost 70,000 long-term CCS reported an overall 5-fold increased risk of developing oral cancer as compared to the general population [15]. Hodgkin lymphoma and leukemia survivors treated with head and neck radiotherapy were at a 33-fold increased risk of developing salivary gland tumors, whereas CCS treated with chemotherapy had a substantially increased risk of tongue cancer (RR = 5.6, 95% CI: 1.0–31.2) [15]. A large study among recipients of allogeneic HCT found that young age at transplantation, total body radiation, and (a history of) cGVHD contributed to an increased risk of oral squamous cell carcinoma [87].

Another subsequent malignancy in CCS involves the thyroid gland, predominantly attributable to radiation exposure, but chemotherapy and immunotherapy may also play a role [88,89,90]. An increased risk for basal cell carcinoma has also been reported, particularly in skin exposed to radiotherapy [91].

Regular (with 6–12 months intervals) screening aimed at early identification of malignancies in the head and neck area is indicated. This could be performed at the late effects clinic, but also dental professionals should be alert in order to detect potential malignancies in the head and neck area. Screening should not be limited to the oral cavity, but should include inspection of the facial skin, as well as inspection and palpation of lymph nodes, salivary glands, and the thyroid gland for enlargement and nodules. Given the increased risk of various cancers, smoking and excessive alcohol use should be discouraged [15,92].

### 3.9. Oral Health Related Quality of Life

Quality of life (QoL) has become an increasingly important outcome measure in oncology. Oral health-related quality of life (OHRQoL) represents QoL in relation to perceived oral health. The subjective evaluation of OHRQoL “reflects people’s comfort when eating, sleeping, and engaging in social interaction; their self-esteem; and their satisfaction with respect to their oral health” [93]. While the overall QoL of CCS has been measured by several studies [94], studies directed to OHRQoL are scarce. One study reported that CCS aged 8–14 years did not perceive a decreased OHRQoL as compared to their peers [95]. A recent study on self-reported outcomes of oral health and OHRQoL in 249 long-term CCS concluded that the perceived OHRQoL was relatively good despite the high prevalence of oral complications [13]. It is not known if non-oral sequelae in CCS affect OHRQoL.

## 4. Late Non-Oral Sequelae That May Affect Oral Health and Provision of Dental Care

CCS have a higher incidence of developing serious health conditions compared to their siblings, and the incidence continues to increase with age [96,97]. These conditions largely relate to aggressive treatments to cure childhood cancer as these therapies generate free radicals and induce DNA damage and telomere attrition [98,99]. The endocrine, pulmonary, cardiac and circulatory, genital, urinary, and nervous systems are the most frequently affected and may contribute to the early onset of common diseases such as diabetes mellitus, cardiovascular disease, hypertension, and secondary cancers [100].

Late complications may not manifest until many years or even decades after completion of cancer treatment [5,101]. Therefore, it is important that CCS are followed in clinics that specialize in early recognition and management of late complications, and that CCS and their caregivers receive information about risks and precautions. Nevertheless, CCS, as well as primary health care providers, including dental professionals, are not always aware of the increased risks for late conditions. It is beyond the scope of this article to address all potential late adverse effects in CCS, but a selection of adverse general health conditions related to radiotherapy and chemotherapy that may affect oral and dental health or may have implications for providing dental care will be briefly discussed.

### 4.1. Endocrine Dysfunction

Endocrine complications are among the most common late effects in CCS, with around 50% of survivors experiencing at least one hormonal disorder later in life [102]. Endocrine-related late effects include dysfunction of the hypothalamic–pituitary axis, thyroid, adrenal, and gonadal dysfunction, type II diabetes mellitus, obesity, metabolic syndrome, and decreased bone mineral density [103,104].

### 4.2. Diabetes Mellitus

CCS have an increased risk of developing diabetes mellitus type II. This risk is further increased among individuals treated at a young age, among those exposed to abdominal radiotherapy or total body irradiation, and those who developed metabolic syndrome [105]. Moreover, both diabetes mellitus and metabolic syndrome may contribute to the risk of cardiovascular disease in CCS.

Poorly controlled or undiagnosed diabetic patients are at risk of poor oral health, including hyposalivation, candidiasis, poor wound healing, and periodontitis. There is a bidirectional relation between diabetes mellitus and periodontal disease: individuals with diabetes are more likely to develop periodontitis and diabetic patients with periodontitis have poorer glycemic control [106,107]. Therefore, controlling these two conditions might have a positive effect on both diseases.

When providing care to diabetes patients, dental providers must keep the patient’s blood glucose levels in mind since hyperglycemia as well as hypoglycemia may lead to emergencies. Dental procedures can cause physiological and psychological stress, which can increase the glucose level. Hypoglycemia is relatively uncommon in patients with type II diabetes, but may be associated with insulin use and other diabetes medications and insufficient intake of carbohydrates [108].

### 4.3. Thyroid Dysfunction

The thyroid gland is regulated by the hypothalamic–pituitary–thyroid axis and produces hormones crucial for cellular metabolism. The pituitary gland secretes thyrotropin (TSH; Thyroid Stimulating Hormone) that stimulates the thyroid to secrete thyroxine (T4) and, to a lesser extent, triiodothyronine (T3). The actions of these hormones include the metabolism of carbohydrates, fats, and proteins, as well as thermoregulation [109]. Thyroid dysfunction is common in CCS, especially in individuals who were treated with head and neck radiotherapy at a young age (inducing damage to the hypothalamic–pituitary axis) or radiotherapy involving cervical, supraclavicular, or mantle fields (direct damage to the thyroid gland).

Thyroid-related complications include hypothyroidism, benign or malignant thyroid tumors, and, rarely, hyperthyroidism [110]. Symptoms of hypothyroidism include fatigue, cold intolerance, dry skin, hair loss, constipation, weight gain, muscle weakness, and bradycardia, whereas hyperthyroidism manifests as thyroid enlargement, irritability, heat intolerance, tremors, increased sweating, hypertension, and tachycardia [109].

Thyroid dysfunction may manifest in any part of the body, including the mouth.

Oral complications of hypothyroidism include macroglossia, delayed eruption, altered tooth morphology, poor periodontal health, delayed wound healing, and increased infection risk [111]. Furthermore, both hypo- and hyperthyroidism have been associated with cardiovascular disease [112,113].

### 4.4. Adrenal Insufficiency

Central adrenal insufficiency may develop in CCS who have been treated with radiation therapy involving the hypothalamic–pituitary axis [102]. As a result, the production of adrenocorticotropic hormone (ACTH), which triggers the adrenal glands to produce cortisol, may be disrupted. Cortisol helps to keep blood glucose levels within normal limits and helps the body to deal with physical stress, such as fever or injury. When a patient with adrenal insufficiency undergoes a surgical procedure, it may be necessary to temporarily increase the dose of glucocorticoids because the adrenal glands are not able to deal with a stressful condition, which may lead to an adrenal crisis [114]. This is a medical emergency that can cause sweating, hypotension, nausea, and circulatory collapse.

### 4.5. Gonadal Dysfunction

Hypogonadism in CCS can be divided into central hypogonadism and primary gonadal failure [115]. An intact hypothalamic–pituitary–gonadal axis is required for normal growth and pubertal development into adolescence. In CCS, central hypogonadism may develop as a result of damage to the hypothalamic–pituitary axis after radiation treatment for brain tumors or by the tumor itself. Cranial irradiation can induce early puberty, especially in girls, as well as delayed puberty since cranial radiotherapy may result in diminished or absent release of luteinizing hormone (LH) and follicle-stimulating hormone (FSH), which leads to hypogonadism and may be accompanied by deficiencies of other pituitary hormones [116].

Primary gonadal insufficiency may develop if cancer treatment damaged the ovaria or testes, resulting in decreased levels of sex steroid hormones (estrogen in women and testosterone in males). Both female and male CCS may be at risk for gonadal insufficiency, resulting in puberty delay or cessation, reduced or delayed growth spurt, reduced bone mass, sexual dysfunction, infertility, and psychosocial problems. In addition, male CCS may present with gynecomastia, diminished muscle mass, and abdominal obesity and female CCS with disrupted menstrual cycles, hot flushes, and sweating. CCS at risk of gonadal dysfunction should be monitored, screened, and counseled by specialized physicians, and treatment often includes hormonal supplementation therapy.

An early or delayed growth spurt may affect the timing of orthodontic treatment. Most studies on the effects of sex hormones on the oral cavity have focused on women of reproductive age as women experience marked increases or decreases in these hormones, while men experience more gradual changes. Endogenous-produced or exogenous-administered sex hormones may trigger physiological responses in the oral tissues, with possible clinical implications, such as gingival inflammation and alterations of the oral microbiome [117]. Low levels of these hormones cause oral mucosal atrophy. Reduced alveolar bone mass has been reported to be associated with periodontitis and a higher risk of implant failure [118,119].

### 4.6. Cardiovascular Disease

CCS are at high risk of developing cardiovascular diseases at an early age. These include coronary artery disease, stroke, and heart failure [120]. Cardiotoxic treatment modalities include drugs such as anthracyclines, high dose alkylating agents, and more recently introduced targeted therapies, as well as irradiation of the heart. Other associated factors for cardiac abnormalities in CCS include younger age at treatment and comorbidities such as high blood pressure, metabolic syndrome, diabetes mellitus, obesity, and an inactive lifestyle [120].

Cardiovascular disease and therapeutics may affect oral health and dental treatment, which may require certain precautions [121]. Periodontitis is considered to have a modulating role in cardiovascular disease, and preventing periodontitis may affect its onset or progression [52,122,123]. There are no oral manifestations directly resulting from cardiovascular disease, but medications used for cardiovascular disease may cause taste changes, stomatitis, gingival bleeding, petechiae, xerostomia, or lichenoid mucosal lesions. Calcium channel blockers may cause gingival overgrowth [124].

### 4.7. Increased Infection Risk

Patients who underwent splenectomy or received radiotherapy affecting the spleen may be at higher risk of serious infections as the spleen is a multifunctional organ that supports the immune system [125]. Antibiotic prophylaxis is not routinely indicated prior to dental procedures for asplenic adult dental patients. However, antibiotic prophylaxis should be considered for children, immunocompromised patients with underlying causative disease, or any patient during the first 3 years after a splenectomy [126].

In addition, CCS suffering from cGVHD have an increased risk of infection due to immunosuppression caused by the disease and its therapy. In these patients, invasive dental interventions should be coordinated with the medical team [56,79].

### 4.8. Mental Health Considerations

Experiences associated with the cancer diagnosis itself or consequences of the treatment may negatively affect the mental health of CCS [127]. In particular, a short stature and craniofacial deformities may negatively affect self-esteem. Although studies reported low or similar stress levels in adolescent CCS compared to the general population, subgroups experience increased psychological stress and symptoms of depression and anxiety [127]. A study directed at dental anxiety in CCS found no elevated levels of anxiety for dental procedures [128].

CCS may experience cognitive dysfunction manifesting months to years after treatment. Risk factors include young age at diagnosis, cranial irradiation, use of methotrexate, female sex, and pre-existing comorbidities. Restricting the use of cranial irradiation reduced the severity of cognitive dysfunction, especially in patients with leukemia. Nevertheless, in particular CCS with a history of primary brain tumors may experience problems with learning new skills, attention, speed of processing, working memory, and visual–motor integration [129]. Cognitively impaired individuals may have a reduced ability to maintain oral health.

## 5. Concluding Remarks and Future Directions

Survivors of childhood cancer are at risk of developing a wide range of adverse late effects in and beyond the orofacial region which may manifest far past the end of cancer treatment. However, cancer treatment regimens vary widely and long-term data derived from large-scale longitudinal studies are still scarce. Therefore, collaborative studies and pooling of data will advance approaches to individually tailored risk-prediction and prevention of late effects in CCS. Future studies should also include assessment of toxicities of novel therapies and genetic risk factors [130].

Manifestations of many late effects may be partially ameliorated by early diagnosis and intervention. Therefore, CCS require extensive follow-up care, preferably in specialized late effects clinics. In addition, health care providers outside the pediatric oncology setting, including dental professionals, can contribute to the care of CCS and improve their QoL [131,132].

CCS (and their caregivers) should be aware of the higher risk of oral and craniofacial complications and the need for regular dental checkups, while dental care providers should be aware of the cancer history of their patients as this may have an impact on their oral health and may necessitate more intensive preventive measures and precautions. Understanding a patient’s medical history is a crucial part of patient assessment when providing dental care to any patient but is especially important in CCS. It would be helpful if health care providers, including dentists and dental specialists, could obtain insight into the individual risk of CCS developing complications.

Therefore, current initiatives to develop cancer treatment summaries and survivorship care plans (“survivorship passports”), including risk-based recommendations for the surveillance of dental problems, disrupted craniofacial growth, and subsequent oral cancer, are very promising and needed [133,134].

To provide optimal and safe dental care, the dentist may need guidance from medical providers about possible contraindications or specific precautions that should be taken prior to invasive dental procedures. Ideally, a survivorship passport should include contact information that facilitates such consultation and enables the dentist to report late oral complications to the late-effects physician.

This article briefly reviewed a number of medical complications of childhood cancer treatment, particularly those that may affect oral health and/or providing dental treatment. Nevertheless, other late complications related to childhood cancer treatment (for example, pulmonary diseases) may also have implications for dental treatment (i.e., surgical interventions, sedation procedures, prescribing medication). Moreover, CCS may use medications, which may have an impact on oral health and may have implications for providing dental care.

Education about oral manifestations of cancer, acute oral complications associated with therapy in childhood cancer patients (e.g., oral mucositis, infection, dysphagia, dry mouth, taste alteration), and late complications in CCS deserves more attention in dental, medical, and nursing curricula and continuing education programs. The importance of multiprofessional collaboration should be emphasized to improve the quality of care. It is also of paramount importance to educate CCS about possible late effects of their specific childhood cancer treatment, including potential oral health effects, and encourage the development of self-management skills.

Although more research is necessary on individual risk factors, it is advisable for dental practitioners to identify CCS in their practice and pay particular attention to early detection of dental and craniofacial growth and developmental disturbances, salivary gland dysfunction, caries, periodontal and oral mucosal diseases, and (peri)-oral malignancies. Furthermore, careful assessment of medical conditions and medications that may impact oral health and dental treatment should be performed.

## Data Availability

Not applicable.
